# Association between axial elongation and corneal topography in children undergoing orthokeratology with different back optic zone diameters

**DOI:** 10.1186/s40662-024-00418-w

**Published:** 2025-01-03

**Authors:** Qi Tan, Randy Kojima, Pauline Cho, Stephen J. Vincent

**Affiliations:** 1 School of Optometry, The Hong Kong Polytechnic University, Hung Hom, Kowloon, Hong Kong SAR; 2 College of Optometry, Pacific University, Oregon, USA; 3 Contact Lens and Visual Optics Laboratory, Optometry and Vision Science, Centre for Vision and Eye Research, Queensland University of Technology, Brisbane, Australia; 4 Department of Optometry and Vision Sciences, West China School of Medicine, Sichuan University, Chengdu, China

**Keywords:** Axial elongation, Orthokeratology, Topographic changes

## Abstract

**Purpose:**

To explore the associations between myopia defocus dosage (MDD), aberration coefficients (primary spherical aberration and coma), and axial elongation in children undergoing orthokeratology (ortho-k) with back optic zone diameters (BOZD) of 5 mm and 6 mm over 2 years.

**Methods:**

Data from 80 participants from two ortho-k studies were analyzed: 22 and 58 children wore lenses with 5-mm and 6-mm BOZD, respectively. Four MDD metrics were calculated from corneal topography data over a 5-mm pupil for the 1-month and 24-month visits: the circumferential, flat, steep, and volumetric MDD. Corneal primary spherical aberration and comatic aberrations were also extracted from topography data over a 5-mm pupil. Linear mixed modelling was performed to explore the associations between the MDD, corneal aberrations, and axial elongation over 2 years, while controlling for confounding factors (e.g., baseline age and sex).

**Results:**

Participants in the 5-mm BOZD group displayed less axial elongation than the 6-mm BOZD group over 2 years (0.15 ± 0.21 mm vs. 0.35 ± 0.21 mm, *P* < 0.001). A greater volumetric MDD was observed in the 5-mm BOZD group compared with the 6-mm BOZD group at the 1- and 24-month visits (both *P* < 0.001). No significant differences were observed between the two groups for the other MDD metrics or corneal aberration coefficients (all *P* > 0.05). Less axial elongation was associated with a greater volumetric MDD at the 1- and 24-month visits (both β = –0.01, *P* < 0.001 and *P* = 0.001), but not with any other MDD metrics or corneal aberrations (all *P* > 0.05).

**Conclusions:**

The volumetric MDD over a 5-mm pupil after 1 month of ortho-k lens wear was associated with axial elongation after 24 months, and may be a useful predictor of future axial elongation in children undergoing ortho-k.

## Background

Of the optical interventions to retard axial elongation, orthokeratology (ortho-k) has been reported to show the greatest efficacy [1], resulting in 43%–63% (0.22–0.36 mm) less axial elongation in myopic children when compared to single-vision spectacles [2–7] or soft contact lenses over 2 years [8]. The ideal topographical outcome of ortho-k for the correction of myopia is a bullseye response to provide quality vision during the daytime [9]. The bulls-eye response is a result of central corneal flattening and mid-peripheral steepening, leading to a corneal power shift (CPS) across the pupil [10]. In theory, the CPS at the pupil margin relative to that of the corneal apex or pupil center, namely the relative CPS, may result in relative peripheral retinal myopic defocus [11–13], which has been suggested as a potential factor underlying the slowing of axial elongation in ortho-k [14]. Consequently, the relative CPS has been used as a surrogate measure of imposed relative peripheral retinal myopic defocus to explore the association between axial eye growth and different ortho-k treatments in myopic children [15–22].

Previous studies have used anterior corneal topographical indices to predict long-term axial elongation in children undergoing ortho-k [15–21]. For example, Zhong et al. reported that the maximum relative CPS along three axes (i.e., nasal, temporal, and inferior) within a 4-mm pupil area after 3 months of ortho-k was significantly associated with axial elongation in Chinese children after 2 years [15]. In contrast, in a study of white European children, the relative CPS (3–5 and 5–8 mm from the corneal apex) were not associated with axial elongation after 2 years of ortho-k treatment [16]. These conflicting results may be due to methodological differences, such as the use of manual and automated topographical analyses, or participant characteristics, such as age, ethnicity, and refractive error. In subsequent studies [17, 18], age was considered a predictive factor in exploring the association between axial elongation and the areal summed relative CPS. The areal summed relative CPS over a central corneal region (4 mm [18] or 7.2 mm diameters [17]) were associated with axial elongation over 1 [18] and 2 years [17], respectively. By definition, the relative CPS is dependent upon the corneal area selected and the number of data points arbitrarily included for summation, making comparisons between studies difficult. For instance, Zhong et al. summed the fitted mean relative CPS of 720 concentric rings within a 7.2-mm diameter cornea, after spline fitting the measured data at 0.01 mm intervals [17]. The summed mean relative CPS was 10.84 ± 5.28 D after 3 months of ortho-k for a 6-mm back optic zone diameter (BOZD) lens [17]. Hu et al. used a different approach and divided the central 4-mm corneal area into 300 annuli (1.2 degrees apart around 360 degrees) and summed the product of the mean relative CPS of each sector and its corresponding area [18]. The mean summed relative CPS $\times$ sector area was 6.90 ± 6.09 D⋅mm^2^ after 1-month ortho-k [18], but the BOZD of the lens used was not specified [18], although this parameter is a key factor which influences the relative CPS profile after ortho-k [22] and affects the rate of axial elongation in children [23]. Since the typical photopic pupil size of children aged 6–12 years is 4.60 ± 0.70 mm [24], it may be more relevant to analyze the relative CPS over a 5-mm corneal diameter to explore the relationship between corneal metrics and axial elongation in children, rather than a smaller or larger corneal diameter (4 mm [18] and 7.2 mm [17]) as considered previously.

Several metrics have also been proposed to describe the corneal location where the relative CPS reaches its peak value [19, 20, 22]. These metrics include the distance from corneal apex (*X*_*max*_) to the maximum relative CPS [20], and the distance between corneal apex (*X*_50_) and the location of half* R*_*max*_ (where *R*_*max*_ is the maximum mean relative CPS of concentric rings) [19, 22]. Yang et al. found that a smaller *X*_50_ after 1-month of ortho-k was associated with less axial elongation over 1 year [19], while Zhang et al. reported that *X*_*max*_ cannot be used to predict long-term axial elongation after controlling for age [19, 20]. While a dose–response relationship may exist between the induced relative CPS and axial elongation, these conflicting results indicate that the location of the maximum CPS may not be the ideal corneal metric to predict future axial elongation.

Currently, it is difficult to determine the optimal CPS dosage associated with less axial elongation using the areal summed relative CPS [17, 18] or the location of maximum relative CPS [19, 20, 22]. In this analysis, a topographical description, the myopia defocus dosage (MDD), was used to quantify the magnitude of relative CPS in selected meridians, at the pupil edge, and in terms of the volume integral over a 5-mm pupil diameter. Other post-ortho-k topographical parameters such as primary spherical aberration and comatic aberrations (vertical coma and horizontal coma) over a 5-mm pupil were also examined, since less axial elongation has been associated with a positive shift in primary spherical aberration [25] and an increase in vertical coma [26] in children undergoing ortho-k. The aim of this study was to determine whether short-term post-ortho-k changes in the MDD and corneal aberrations over a 5-mm pupil were associated with longer-term (24 months) axial elongation after controlling for confounding factors (e.g., baseline age).

## Methods

### Participants

Data was retrieved for 35 and 45 ortho-k participants who completed the 2-year Atropine Combined with Orthokeratology (AOK) Study [27] and the Variation of Orthokeratology Lens Treatment Zone (VOLTZ) Study [28], respectively. The study designs have been reported in detail previously [27, 28]. In brief, participants were children of Chinese ethnicity, ages 6 to less than 11 years, with normal ocular health and myopia of 1.00–4.00 D inclusive in the AOK study [27]; and 0.75–4.00 D inclusive in the VOLTZ study [28]. The same lens fitting and replacement rationale were followed in the AOK and VOLTZ studies [27, 28]. In the VOLTZ study, 22 and 23 participants were randomized to wear ortho-k lenses with BOZD 5 mm and 6 mm (KATT BE Free Lens, Precision Technology Services, Vancouver, B.C., Canada), respectively. All of the 35 participants from the AOK study wore the KATT BE Free lens alone with a BOZD of 6 mm. None of the participants included in this analysis were treated with atropine. Only data from the right eyes were used for analyses.

### Measurements

The AOK and VOLTZ studies were performed at the Optometry Clinic of the School of Optometry at The Hong Kong Polytechnic University. All participants underwent cycloplegic examinations (two drops of 1% cyclopentolate administered 5 min apart) after 1 month and every 6 months after commencement of ortho-k lens wear. Ethics approvals for both AOK (HSEARS20160406005) and VOLTZ (HSEARS20170118004) studies were obtained from the Human Subject Ethics Subcommittee of the School of Optometry of The Hong Kong Polytechnic University. For the AOK study, ethics approval was also obtained from the Institutional Review Board of The University of Hong Kong (HKU)/Hospital Authority Hong Kong West Cluster. All children provided assent and parents provided informed consent before participation, with all procedures following the tenets of the Declaration of Helsinki.

Axial length was measured by a masked examiner using the IOLMaster (Carl Zeiss Meditec AG, Jena, Germany) at least 30 min after cycloplegia. Manifest subjective refractive error was measured using a trial frame after cycloplegia, following the principle of maximum plus for maximum visual acuity (VA). Best-corrected visual acuity (BCVA) was measured using high contrast (100%) Early Treatment Diabetic Retinopathy Study charts (Precision Vision, La Salle, Illinois, USA) under normal room lighting at a 4-m distance. Corneal topography was measured using the E300 corneal topographer (Version 6.1.2; Medmont Pty. Ltd., Nunawading, Australia). Four maps with a score of at least 98 were captured for each eye and used for analysis. A subtractive tangential power map was used to evaluate lens centration after overnight lens wear by a trained clinical assistant. Only the data of participants with a bullseye response (a complete ring of topographical change with lens decentration ≤ 1 mm [28, 29]) were included for analyses. For each subject, all measurements were made by the same examiners at every visit during the study period.

### Assessment of topographical parameters

**Fig. 1:**
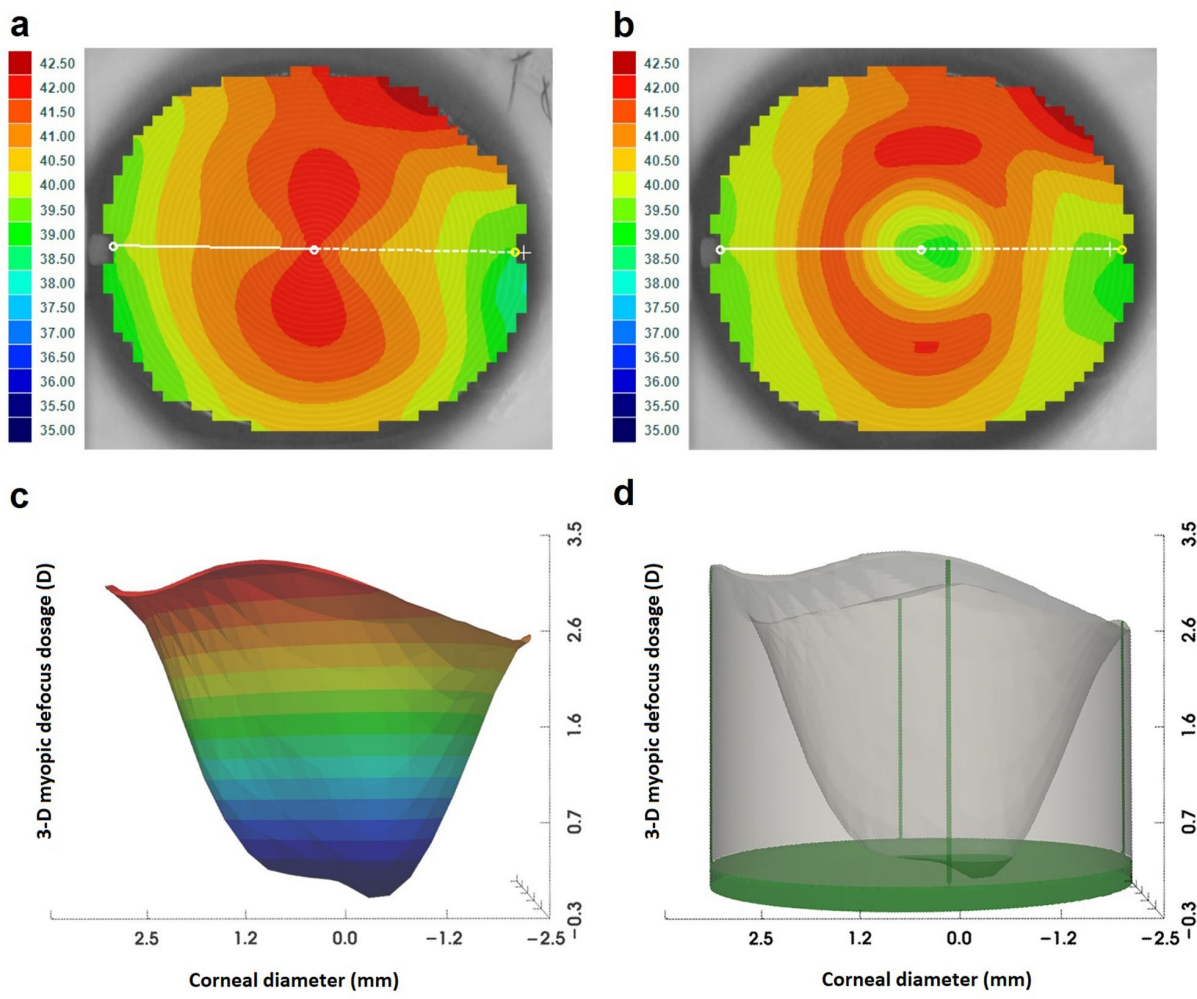
An example of volumetric myopic defocus dosage (3-D MDD) over a 5-mm corneal diameter for a participant wearing an orthokeratology (ortho-k) lens with a back optic zone diameter of 5 mm. **a** Pre-ortho-k axial power map; **b** Post-ortho-k axial power map; **c** 3-D illustration of normalized relative corneal power shift (CPS), using color bar indicating different values; **d** Perspective view of 3-D MDD; the smallest negative value is − 0.25 D, indicating that power of corneal apex is 0.25 D flatter than that at map center after ortho-k. The volume above the flat surface of the map center is depicted as grey, while that under is depicted green. The resulting 3-D MDD is 44.34 D·mm^2^

**Fig. 2:**
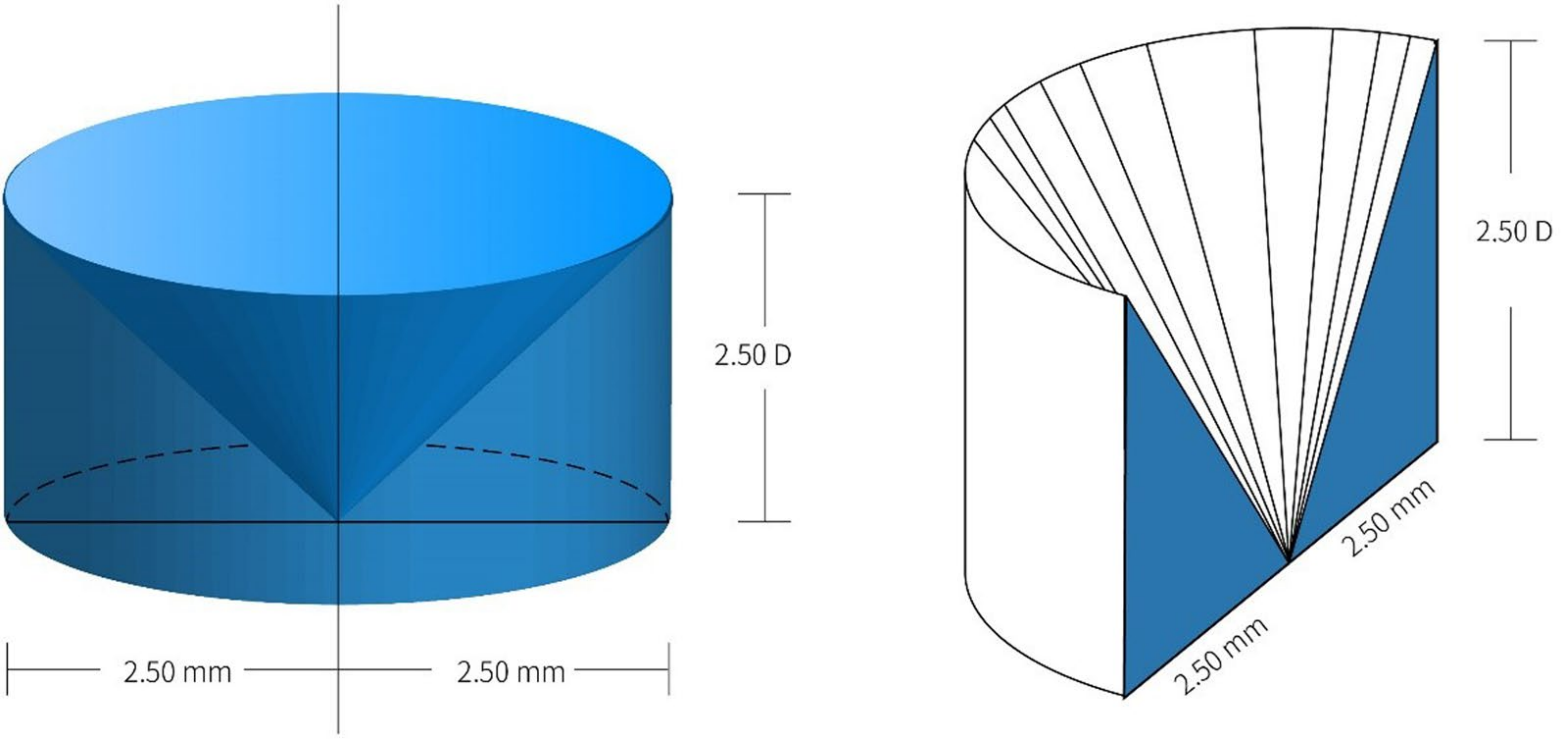
Illustration of fitted circumferential MDD of 2.50 D for volumetric myopic defocus dosage of 32.72 D·mm^2^

Anterior corneal wavefront aberrations were calculated from the topography maps using a 4th-order Zernike expansion over a 5-mm corneal diameter. Zernike terms of primary spherical aberration, vertical coma, and horizontal coma were extracted for analysis. The CPS was calculated on the Medmont E300 software platform by comparing baseline (pre-ortho-k) and post-ortho-k topographical maps. In line with previous studies [15–22], the axial map was used to define CPS, since it is more resistant to noise than tangential maps [30]. Using axial power maps, the CPS within a 5-mm pupil area was first normalized to the power change at the center of the axial power map. While the corneal apex does not always align with the center of an axial map, after normalization, the corneal apex was located at the data point demonstrating the smallest negative value, as the largest reduction in power was expected to occur at the corneal apex after ortho-k [31]. The difference between the normalized apical corneal power change and that at the 5-mm margin provided the relative CPS, namely the two-dimensional (2-D) MDD. Three 2-D independent MDD were calculated: (1) circumferential MDD: mean of 32 points around the circumference of a 5-mm pupil 11.25° apart; (2) flat MDD: mean of the relative CPS of the two opposing points at the 5-mm pupil margin along the flat corneal meridian; (3) steep MDD: mean of the relative CPS of two opposing points at the 5-mm pupil margin along the steep corneal meridian. Using a spline fit, the curve of relative CPS along 360 meridian was fitted using 0.01 mm increments. The volumetric MDD, namely the 3-D MDD over a 5-mm corneal diameter, was calculated as the volume integral of the 3-D space surrounded by the curved surface of the relative CPS and the reference plane (i.e., the plane of the corneal apex), as shown in Fig. 1, which demonstrates the volumetric MDD using a 5-mm BOZD ortho-k lens. The fitted circumferential MDD was calculated for each volumetric MDD, assuming that the relative CPS around the 5-mm pupil margin was evenly distributed (Fig. 2), using the following formula:

$$fitted\, circumferential \,MDD = \frac{{Volumetric \,MDD \times {\raise0.7ex\hbox{$3$} \!\mathord{\left/ {\vphantom {3 2}}\right.\kern-0pt} \!\lower0.7ex\hbox{$2$}}}}{{\pi \times \left( {2.5\, mm} \right)^{2} }}$$

As topographical changes stabilize within 1 month after commencing ortho-k [32], changes in topographical parameters after 1 month post-ortho-k were used to examine the association with axial elongation over 2 years.

### Statistical analysis

Analyses were performed using IBM SPSS Statistics for Windows (version 25.0. IBM Corp., Armonk, New York, USA). The normality of the data was determined using the Kolmogorov–Smirnov test. Crosstab analysis was used to compare the sex ratio. Unpaired *t*-tests were used to compare baseline characteristics [age, cycloplegic spherical equivalent refraction (SER), axial length, BCVA, and topographical parameters], post-ortho-k changes in topographical parameters, and changes in axial length, between the two ortho-k groups (the 5- and 6-mm BOZD groups). As the corneal coefficients of primary spherical aberration, vertical coma, and horizontal coma were not normally distributed, Wilcoxon signed-ranks tests were used to analyze these data. A linear mixed model (Model 1) was used to assess the association between predictor variables (baseline age, sex, cycloplegic SER, BCVA, axial length) and axial elongation, with a first-order autoregressive covariance structure and restricted maximum likelihood estimation. Individual slopes and intercepts were included as random effects, using an unstructured covariance matrix to control inter-subject variation. If any baseline parameters were significantly associated with the 2-year axial elongation in Model 1, they were included in Models 2–5. The association between MDD and changes in aberrations at the 1-month visit, the changes in MDD over time, and changes in aberrations over time, with the 2-year axial elongation were examined in Models 2–5 using the same structure as Model 1. A *P* value less than 0.05 indicates statistical significance.

## Results

Data from 80 participants were analyzed: 22 wore ortho-k lenses with BOZD 5-mm (the 5-mm group), and 58 children wore ortho-k lenses with BOZD 6-mm (the 6-mm group). Before pooling the VOLTZ and AOK data for the 6-mm group, baseline demographic and clinical characteristics were compared between AOK and VOLTZ participants wearing 6-mm BOZD lenses. No significant differences were observed, except for cycloplegic SER, which was on average 0.56 D more myopic in the AOK participants (AOK: − 2.83 ± 1.01 D; VOLTZ: − 2.27 ± 0.85 D, *P* = 0.03). After pooling, the baseline demographics and data of the 5-mm and 6-mm groups are summarized in Table 1. Except for BCVA, no significant differences in age, sex, cycloplegic SER, axial length, or topographical parameters were found between the two groups at the baseline visit (all *P* > 0.05). The difference in BCVA was less than two letters (i.e., 0.04 logMAR), indicating that the difference was clinically insignificant [33]. After ortho-k, the 2-D MDD (circumferential, flat, and steep MDD) and the changes in corneal aberrations (spherical aberration, vertical and horizontal coma) were not significantly different between the two groups (all *P*s > 0.05) (Table 2). The volumetric MDD and the fitted circumferential MDD were significantly greater in the 5-mm group compared to the 6-mm group at 1-month and 24-month visits (both *P* < 0.001, Table 2). After 2 years of treatment, the mean ± SD axial elongation in the 5-mm group (0.15 ± 0.21 mm) was significantly less than that in the 6-mm group (0.35 ± 0.21 mm, *P* < 0.001). Since a significant association between baseline age and axial elongation over 2 years was observed (Model 1) and more rapid eye growth has been previously observed in 6–8 year olds than 9–11 year olds treated with ortho-k [3], axial elongation was also compared between these two age groups using unpaired *t*-tests (Table 3). For the 6-mm group, participants aged 6–8 years demonstrated more rapid eye growth than those aged 9–11 years (*P* = 0.03; Table 3), while no significant difference with respect to age was observed in the 5-mm group (*P* = 0.11, Table 3).

**Table 1:** Baseline demographics and data (mean ± standard deviation or median [min, max]) of participants retrieved from the AOK and VOLTZ studies

Characteristics	5-mm BOZD	6-mm BOZD	*P*
Number of participants	22	58	–
Age (years)	9.1 ± 1.2	9.2 ± 1.1	0.75
Sex (Female/Male)	13/9	37/21	0.83
Axial length (mm)	24.65 ± 0.79	24.40 ± 0.84	0.40
Cycloplegic SER (D)	− 2.69 ± 0.89	− 2.61 ± 0.98	0.75
BCVA (logMAR)	0.00 ± 0.05	− 0.03 ± 0.05	**0.02**
Primary spherical aberration (µm)	0.18 [0.10, 0.41]	0.19 [0.04, 0.33]	0.83
Vertical coma (µm)	− 0.03 [− 0.22, 0.47]	− 0.05 [− 0.41, 0.48]	0.84
Horizontal coma (µm)	− 0.08 [− 0.25, 0.05]	− 0.10 [− 0.26, 0.09]	0.91

*AOK* = combined 0.01% atropine and orthokeratology; *BOZD* = back optic zone diameter; *VOLTZ* = variation of orthokeratology lens treatment zone; *SER* = spherical equivalent refraction; *BCVA* = best-corrected visual acuity

*P,* probability value of unpaired *t-*tests, Wilcoxon signed-ranks tests, and Crosstab analyses between the two groups

Bold font indicates statistical significance

**Table 2:** Post-ortho-k changes in topographical parameters (mean ± standard deviation) of participants retrieved from the AOK and VOLTZ studies

Parameter	5-mm BOZD (n = 22)	6-mm BOZD (n = 58)	*P*
1-month			
Change in primary spherical aberration (µm)	0.70 ± 0.44	0.57 ± 0.30	0.16
Change in vertical coma (µm)	0.67 ± 0.64	0.43 ± 0.52	0.17
Change in horizontal coma (µm)	0.36 ± 0.72	0.44 ± 0.52	0.78
Circumferential MDD	3.33 ± 1.05	3.15 ± 1.61	0.62
Flat MDD (D)	3.56 ± 1.30	3.32 ± 1.71	0.57
Steep MDD (D)	3.11 ± 0.84	3.02 ± 1.55	0.80
Volumetric MDD (D mm^2^)	45.19 ± 13.28	32.88 ± 10.92	**< 0.001**
Fitted circumferential MDD (D)	3.45 ± 1.01	2.51 ± 0.83	**< 0.001**
24-month			
Change in primary spherical aberration (µm)	0.65 ± 0.49	0.61 ± 0.29	0.89
Change in vertical coma (µm)	0.65 ± 0.58	0.41 ± 0.62	0.31
Change in horizontal coma (µm)	0.59 ± 0.59	0.48 ± 0.51	0.62
Circumferential MDD	3.53 ± 1.23	3.34 ± 1.29	0.55
Flat MDD (D)	3.57 ± 1.40	3.33 ± 1.19	0.45
Steep MDD (D)	3.47 ± 1.19	3.27 ± 1.76	0.63
Volumetric MDD (D mm^2^)	50.63 ± 16.89	37.42 ± 12.51	**< 0.001**
Fitted circumferential MDD (D)	3.87 ± 1.29	2.86 ± 0.96	**< 0.001**

*AOK* = combined 0.01% atropine and orthokeratology; *BOZD* = back optic zone diameter; *VOLTZ* = variation of orthokeratology lens treatment zone; *MDD* = myopia defocus dosage

*P,* probability value of one-way ANOVA for the comparison between the three treatment groups

Bold font indicates statistical significance

**Table 3:** Comparison of axial elongation (mean ± standard deviation) between the 6–8 and 9–11 years age groups over two years

Subgroups	5-mm BOZD (n = 22)	6-mm BOZD (n = 58)	*P*
6–8 years	0.09 ± 0.17 n = 9	0.43 ± 0.19 n = 23	**0.03**
9–11 years	0.24 ± 0.25 n = 13	0.30 ± 0.21 n = 35	0.41
*P*^†^	0.11	**0.02**	

*BOZD* = back optic zone diameter

*P*, probability value of unpaired *t*-test for the comparison between the 5-mm and 6-mm groups

^†^*P*, probability value of unpaired *t*-test for the comparison between the two age groups

Bold font indicates statistical significance

Linear mixed model analyses revealed that axial elongation over 2 years was not associated with any baseline parameters except baseline age (β =  − 0.02, *P* = 0.003) (Model 1, Table 4). Consequently, baseline age was included in all subsequent models. Models 2–4 revealed that axial elongation over 2 years was associated with the volumetric MDD at the 1-month visit (β =  − 0.01, *P* < 0.001; Model 3), and the change in the volumetric MDD over time (β =  − 0.01, *P* < 0.001; Model 5), but not with any other parameters (all *P* > 0.05).

**Table 4:** Linear mixed modelling for the effect of topographical parameters on axial elongation over two years

Participants retrieved from the AOK and VOLTZ studies (n = 80)		
	β	*P*
Model 1		
Intercept	0.00	0.17
Baseline age	− 0.02	**< 0.01**
Time	0.02	**< 0.001**
Baseline cycloplegic SER	− 0.02	0.64
Baseline axial length	0.00	0.56
Baseline BCVA	0.00	0.41
Sex	0.00	0.09
Model 2—Change in aberration at 1-month visit		
Intercept	0.12	0.30
1-month change in spherical aberration	0.02	0.73
1-month change in vertical coma	− 0.02	0.52
1-month change in horizontal coma	0.00	0.34
Model 3—MDD at 1-month visit		
Intercept	0.00	0.08
Circumferential MDD	0.00	0.10
Flat MDD	0.00	0.07
Steep MDD	0.00	0.10
Volumetric MDD	− 0.01	**< 0.001**
Model 4—Change in aberration over time		
Intercept	− 0.06	0.79
Change in spherical aberration	− 0.03	0.17
Change in vertical coma	0.02	0.12
Change in horizontal coma	0.01	0.79
Model 5—MDD over time		
Intercept	0.05	0.61
Circumferential MDD	0.00	0.99
Flat MDD	− 0.01	0.61
Steep MDD	0.02	0.31
Volumetric MDD	− 0.01	**0.001**

*AOK* = combined 0.01% atropine and orthokeratology; *VOLTZ* = variation of orthokeratology lens treatment zone; *SER* = spherical equivalent refraction; *BCVA* = best-corrected visual acuity; *MDD* = myopia defocus dosage

β, estimates for fixed effects in linear mixed modelling; *P*, probability value of association between parameters and axial elongation over two years; Bold font indicates statistical significance

## Discussion

In this study, the relative CPS, following ortho-k treatment with BOZD 5-mm and 6-mm lenses, was described using MDD metrics, based on data retrieved from the 2-year AOK and VOLTZ studies. Significantly less axial elongation was found in the 5-mm group than the 6-mm group over 2 years (0.15 ± 0.21 mm vs. 0.35 ± 0.21 mm, *P* < 0.001). A greater volumetric MDD was also observed in the 5-mm group compared to the 6-mm group over 2 years (*P* < 0.001), indicating greater relative CPS in the 5-mm group. Linear mixed modelling revealed that a greater volumetric MDD (i.e., both 1-month and 24-month values) was associated with less axial elongation in post-ortho-k participants over 2 years (both *P* < 0.001), when controlling for baseline age. Based on this association, the short-term (1-month) volumetric MDD over a 5-mm pupil may be used as a predictor of future axial elongation in Chinese children undergoing ortho-k.

To facilitate the interpretation of the between-group difference in the volumetric MDD, the fitted circumferential MDD was calculated for each given volumetric MDD. The fitted circumferential MDD at the 1-month visit was approximately 1.00 D greater in the 5-mm group than the 6-mm group (*P* < 0.001, Table 2). In comparison, the magnitude of direct circumferential MDD, equivalent to the mean relative CPS of 32 sample points around the circumference of a 5-mm pupil, was not different between the two groups (*P* > 0.05). A paired *t*-test revealed that, in the 6-mm group, the fitted circumferential MDD at the 1-month visit was on average 0.82 D less than the direct circumferential MDD (2.51 ± 0.83 D vs. 3.15 ± 1.61 D, *P* = 0.001). However, this difference was not observed in the 5-mm group (3.45 ± 1.01 D vs. 3.33 ± 1.05 D, *P* = 0.10). These results suggest that the fitted circumferential MDD may be more sensitive than the direct circumferential MDD in terms of the level of imposed volumetric MDD. This study presents another parameter of interest (i.e., the fitted circumferential MDD) that could be used to determine the optimal/minimum dosage of relative CPS to retard axial growth for researchers and practitioners. Based on the between-group difference of approximately 1.00 D in the fitted circumferential MDD, it is recommended that, on average a relative CPS of 4.5 D and 3.5 D at a 5-mm pupil margin, should be induced for ortho-k with 6-mm and 5-mm BOZD, respectively. In terms of volumetric MDD, approximately, a volume of 59.0 D·mm^2^ and 46.0 D·mm^2^ should be induced for ortho-k with 6-mm and 5-mm BOZD, respectively, if targeting an optimal retardation effect. Thus, an additional function could be added to topographers to facilitate the calculation of the volumetric MDD and the fitted circumferential MDD to guide ortho-k lens modifications [15–22]. In addition, the greatest direct circumferential MDD at 1-month visit was found to be 5.37 D and 5.36 D in the 6-mm and 5-mm groups, respectively, suggesting a limitation in relative CPS at the 5-mm pupil margin for the correction of myopia not more than 4.00 D.

While less axial elongation was observed in the 5-mm group compared to the 6-mm group in the VOLTZ study over 2 years (0.15 ± 0.21 mm vs. 0.35 ± 0.23 mm, *P* = 0.005) [23], the magnitude of relative CPS between groups was not examined. The current analysis of the pooled data from the AOK and VOLTZ study revealed that a greater volumetric MDD was generated from the smaller BOZD lens, and a significant negative association was observed between the 1-month volumetric MDD and axial elongation over 2 years. In the VOLTZ study, the treatment zone diameter was on average 1.15 mm smaller horizontally and 0.77 mm smaller vertically in the 5-mm group compared to the 6-mm group [23]. There were also greater increases in ocular higher-order primary spherical aberration and coma (i.e., vertical and horizontal combined) for a 4-mm pupil, in the 5-mm group compared to the 6-mm group, and axial elongation was associated with higher levels of primary spherical aberration [34]. Together, these findings suggest that the level of myopic defocus and higher-order aberrations are major factors influencing axial elongation in ortho-k.

Previous studies have also explored the relationship between ocular aberrations and axial elongation in ortho-k with a conventional 6-mm BOZD [25, 26], but not specifically comparing BOZD 5- versus 6-mm lens design. These studies have shown that less axial elongation has been associated with a positive shift in ocular primary spherical aberration [25] and an increase in vertical coma [26] in children undergoing ortho-k. However, in the current study, changes in corneal primary spherical aberration, and horizontal and vertical coma, were not associated with axial elongation over 2 years (all *P* > 0.05). The failure to observe associations between metrics of higher-order aberration and axial elongation may be due to the use of corneal aberrations (current study) instead of ocular aberrations (previous studies). A recent study by Li et al. investigated the influence of different BOZD (5 mm vs. 6.2 mm) on relative CPS and corneal higher-order aberrations over 1 year [22]. A significantly greater areal summed relative CPS over a 4.8-mm corneal diameter and a greater increase in horizontal coma were observed in the 5-mm BOZD group [22]. However, since the two different BOZD lenses used by Li and co-workers were not of the same brand [22], it is possible that other lens design factors, other than BOZD, may have contributed to the differences in corneal aberrations and the relative CPS, and consequently, different rates of axial elongation.

A limitation of the current study is that only corneal metrics for a fixed 5-mm pupil diameter, such as the volumetric MDD and corneal higher-order aberrations, were analyzed. Ideally, the actual pupil diameter of each individual eye should be considered as this will impact the volume of defocus experienced [35]. Both anterior corneal optics and the retinal shape may contribute to the peripheral refraction and ocular aberrations, and large variations in the retinal shape have been observed among myopes [36]. However, the magnitude of total ocular higher-order aberrations or myopic defocus imposed on the retina were not quantified in the current study, which may limit the ability to predict axial eye growth. The combination of corneal metrics and other ocular measurements (e.g., ocular higher order aberrations and peripheral refraction) could be investigated in future studies to investigate if and how they interact to possibly influence post-ortho-k axial elongation. Another limitation is that treatment zone characteristics were not analyzed or other metrics describing the change in relative CPS [19–22] were not calculated, although these metrics may not reflect the overall dosage of relative CPS after ortho-k. Also, since the results in the current analysis were only derived from topographical changes induced by the KATT BE Free lens, further study is required to confirm translation to other lens designs. Finally, the causal relationship between myopic defocus induced from the anterior cornea after ortho-k and axial elongation was not confirmed in this study. Future work to quantify the modification of myopic defocus resulted from ortho-k are warranted.

## Conclusions

In conclusion, the relative CPS following ortho-k with BOZD 5-mm and 6-mm lenses was described using MDD metrics for a 5-mm pupil. A greater volumetric MDD was observed in the 5-mm group, and a greater volumetric MDD at the 1-month visit was associated with less axial elongation over 2 years, after controlling for baseline age. Consequently, the volumetric MDD after 1-month of ortho-k may be a useful predictor of longer-term axial growth in Chinese children undergoing ortho-k.

## Data Availability

The data supporting the findings of this study are available within the article, and supplementary material can be shared upon request.

## Abbreviations

Ortho-k: Orthokeratology
CPS: Corneal power shift
SD: Standard deviation
BOZD: Back optic zone diameter
MDD: Myopia defocus dosage
AOK: Atropine combined with orthokeratology
VOLTZ: Variation of orthokeratology lens treatment zone
VA: Visual acuity
BCVA: Best-corrected visual acuity
2-D: Two-dimensional
3-D: Three-dimensional
SER: Spherical equivalent refraction
